# Fare Thee Well

**Published:** 1911-03

**Authors:** 


### Fare Thee Well.

(*The Southern Clinic.*)

[Professor Metchnikoff recommends the removal of the large intestine as a means of prolonging life.]

Fare thee well! And if forever,
  Large intestine, fare thee well!
Metchnikoff declares that I can
  Do without thee just as well.
Furthermore, he says, without thee,
  I shall live a longer life—
Hurry with the anesthetic,
  Hasten with the carving knife!

Soon, O useless large intestine,
  Where the germ of age doth grow,
You may meet with the appendix
  That I lost some time ago.
In the wondrous realm of science
  Such astounding things befall—
Soon it may become the fashion
  To have no inside at all.

—*Physicians' Chem. and Drug Journal, Chicago.*

